# Descripción clínico-radiológica de un angiosarcoma cardiaco con metástasis cerebral que simula un quiste hidatídico

**DOI:** 10.7705/biomedica.4335

**Published:** 2019-09-01

**Authors:** Blair Ortiz, Carolina Hernández, Norma Carolina Barajas

**Affiliations:** 1 Grupo de Neurología Infantil, Universidad de Antioquia; Hospital San Vicente Fundación, Medellín, Colombia Grupo de Neurología Infantil, Universidad de Antioquia Hospital San Vicente Fundación Medellín Colombia; 2 Facultad de Medicina, Universidad de Antioquia, Medellín, Colombia Universidad de Antioquia Facultad de Medicina Universidad de Antioquia Medellín Colombia

**Keywords:** hemangiosarcoma, equinococosis, convulsiones, hipertensión intracraneal, Hemangiosarcoma, echinococcosis, seizures, intracranial hypertension

## Abstract

Los angiosarcomas son sarcomas malignos que se originan en las células endoteliales vasculares. Su diagnóstico diferencial es muy amplio debido a su parecido con otras enfermedades, como las parasitarias, y usualmente es un diagnóstico por exclusión. La neurocisticercosis y la hidatidosis cerebral son parasitosis intestinales que pueden comprometer el sistema nervioso central y tienen mayor incidencia en los países suramericanos.

El diagnóstico se establece a partir del perfil epidemiológico, el estudio parasitológico, la apariencia radiológica de las lesiones y el estudio de histopatología del espécimen.

Se presenta el caso de una adolescente con factores de riesgo para parasitosis y neuroimágenes sugestivas de hidatidosis cerebral, cuyo diagnóstico definitivo fue angiosarcoma cardiaco metastásico.

## Abstract (en)

Angiosarcoma is the most malignant sarcoma originating in endothelial vascular cells. It has a wide differential diagnosis due to its similarities with other entities, such as parasitic diseases. More often, angiosarcoma is diagnosed by exclusion. Neurocysticercosis and hydatid disease, or echinococcosis, are parasitic infections that may involve the central nervous system and their incidence is higher in South American countries.

Diagnosis is established based on the epidemiological profile, the parasitological examination, the radiological appearance of the lesions, and the histopathology analysis of specimens.

We present the case of a female adolescent with parasitosis risk factors whose neuroimages suggested cerebral hydatid cysts and who was finally diagnosed with cardiac metastatic angiosarcoma.

Las lesiones quísticas cerebrales se presentan con una sintomatología y un síndrome neurológico muy similares, dependiente del efecto de masa y la ocupación de espacio e independiente de la etiología.

El diagnóstico diferencial es amplio e incluye enfermedades neoplásicas, vasculares e infecciosas, siendo las más importantes las ocasionadas por parásitos. El diagnóstico y el tratamiento representan todo un reto clínico.

La hidatidosis cerebral o equinococosis es una parasitosis infrecuente en Colombia, y la neurocisticercosis es una infección de mayor prevalencia, endémica en ciertas regiones plenamente identificadas por sus antecedentes culturales y de salubridad.

Se presenta el caso de una adolescente con síndrome de hipertensión endocraneana crónica, convulsiones motoras generalizadas y lesiones nodulares en el miembro inferior izquierdo. En la imagenología cerebral se observaron lesiones quísticas gigantes que sugerían la presencia de parásitos intestinales. Sin embargo, en el estudio de histopatología de las lesiones cerebrales y de la extremidad se confirmó un angiosarcoma.

Se discuten las consideraciones diagnósticas y terapéuticas relevantes, así como el potencial parecido clínico con otras enfermedades más frecuentes.

## Presentación de caso

Una adolescente de 14 años de edad procedente de la zona urbana del municipio de El Bagre, departamento de Antioquia, fue evaluada en el Servicio de Urgencias por presentar un cuadro clínico de cuatro meses de evolución con cefalea diaria grave, opresiva, de localización posterior, predominantemente matutina y de varias horas de duración, con vómito en proyectil, diplopía y con antecedentes de dos convulsiones motoras de inicio no presenciado con propagación bilateral.

La paciente procedía de una región de la periferia ribereña, con condiciones higiénicas precarias, endémica para parasitosis intestinales, desnutrición y tuberculosis.

En el examen neurológico se encontró una agudeza visual de 20/200, con percepción de la luz, el color y el movimiento, con hemianopsia homónima contralateral derecha, papiledema bilateral, vasos retinales ingurgitados, escasas hemorragias petequiales peripapilares, y dos nódulos semiduros y móviles en el miembro inferior izquierdo y riesgo nutricional.

**Figura 1:**
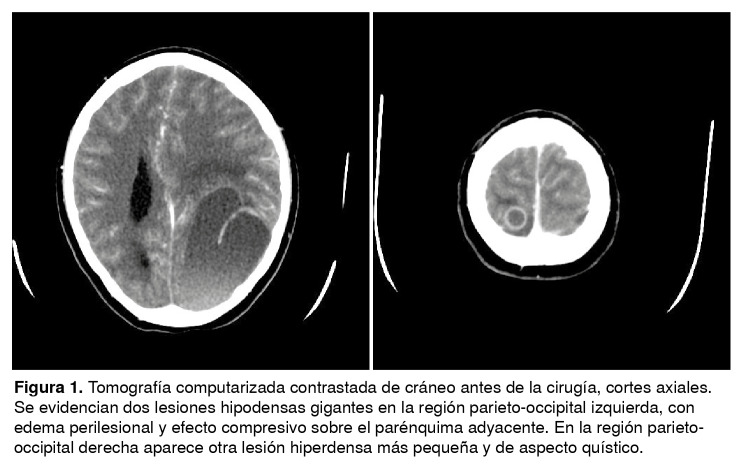
Tomografía computarizada contrastada de cráneo antes de la cirugía, cortes axiales. Se evidencian dos lesiones hipodensas gigantes en la región parieto-occipital izquierda, con edema perilesional y efecto compresivo sobre el parénquima adyacente. En la región parieto- occipital derecha aparece otra lesión hiperdensa más pequeña y de aspecto quístico.

Se le diagnosticó un síndrome de hipertensión endocraneana, probablemente secundario a una lesión que ocupaba el espacio occipital izquierdo, y convulsiones sintomáticas agudas. En la tomografía computarizada de cráneo se reportaron lesiones de aspecto quístico en la región parieto-occipital izquierda, con edema perilesional y efecto compresivo sobre el parénquima adyacente (figura 1). Se le practicó una craniectomía y se obtuvieron biopsias por incisión.

**Figura 2:**
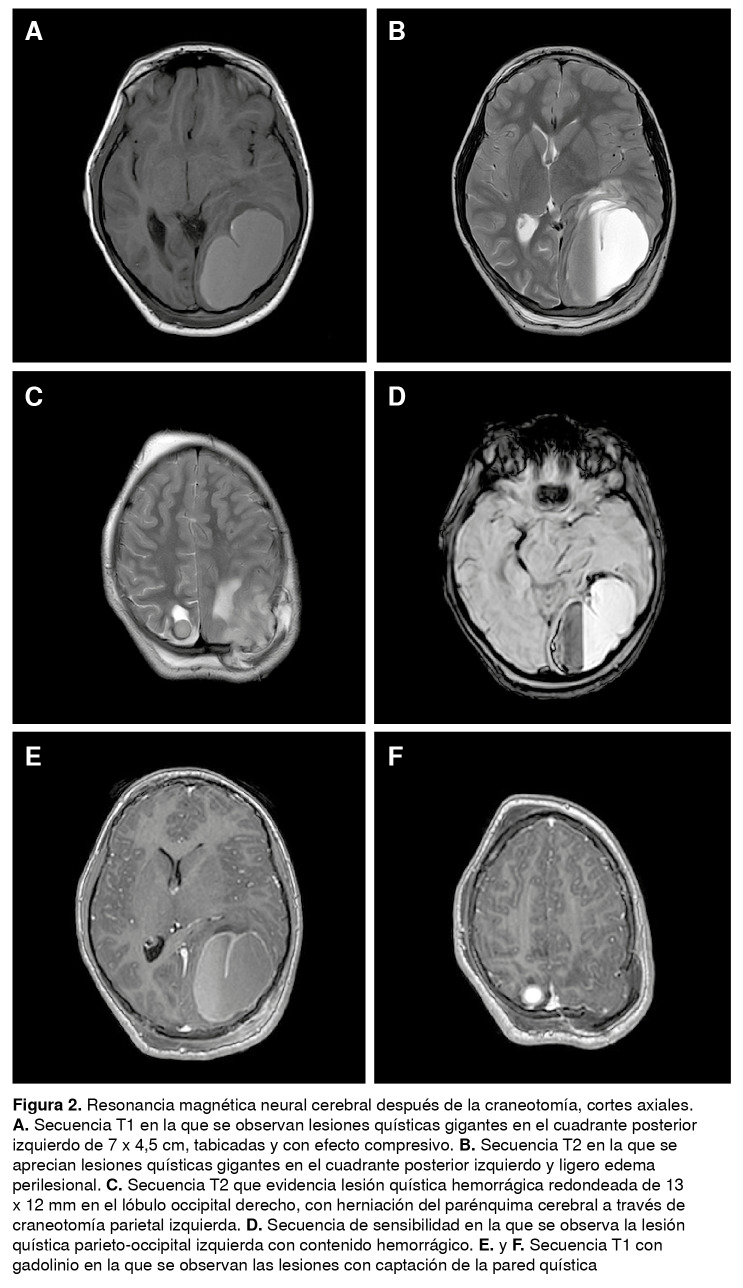
Resonancia magnética neural cerebral después de la craneotomía, cortes axiales.**A.** Secuencia T1 en la que se observan lesiones quísticas gigantes en el cuadrante posterior izquierdo de 7 x 4,5 cm, tabicadas y con efecto compresivo. **B.** Secuencia T2 en la que se aprecian lesiones quísticas gigantes en el cuadrante posterior izquierdo y ligero edema perilesional. **C.** Secuencia T2 que evidencia lesión quística hemorrágica redondeada de 13 x 12 mm en el lóbulo occipital derecho, con herniación del parénquima cerebral a través de craneotomía parietal izquierda. **D.** Secuencia de sensibilidad en la que se observa la lesión quística parieto-occipital izquierda con contenido hemorrágico. **E.** y **F.** Secuencia T1 con gadolinio en la que se observan las lesiones con captación de la pared quística

Se ordenó una resonancia magnética cerebral simple y contrastada en la que se observaron tres lesiones quísticas en los cuadrantes posteriores, la mayor de ellas en la región parieto-occipital izquierda (figura 2). En las secuencias de espectroscopia se conservaba la relación entre colina y N-acetil aspartato, sin picos de colina ni de lactato en algunos vóxeles, lo que sugería la presencia de una condición no neoplásica.

Bajo la sospecha de parasitosis cerebral, se solicitaron exámenes de Western Blot y ELISA para la detección de cisticercos, con resultados negativos, así como un examen coprológico directo y por concentración seriada que no revelaron parásitos intestinales, y el estudio de histopatología de las biopsias tomadas en el miembro inferior izquierdo. En las biopsias de cerebro y de la extremidad izquierda se reportó un tumor de alto grado de malignidad, con tinción positiva para el tipo de sarcoma.

Mediante la ecocardiografía se detectó una lesión intracavitaria infiltrante del ventrículo izquierdo y con la angiorresonancia cardiaca, una masa isointensa en el músculo en las secuencias de cine, hiperintensa en T2, y con áreas de restricción de la difusión y captación heterogénea del contraste en las imágenes tempranas y tardías (figura 3)

**.Figura 3:**
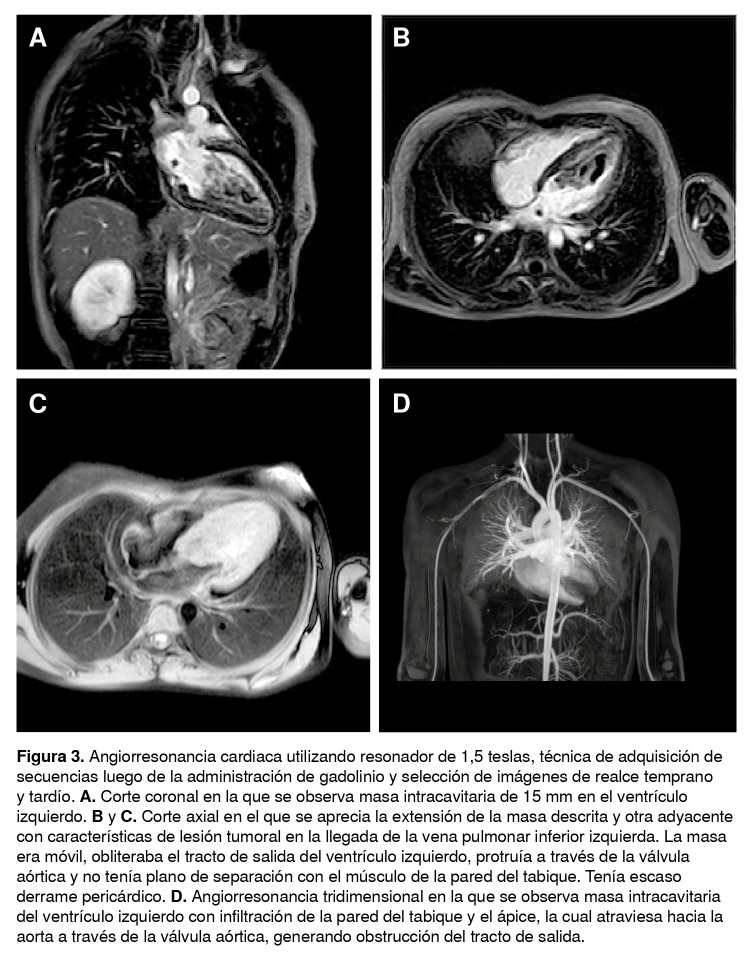
Angiorresonancia cardiaca utilizando resonador de 1,5 teslas, técnica de adquisición de secuencias luego de la administración de gadolinio y selección de imágenes de realce temprano y tardío. **A.** Corte coronal en la que se observa masa intracavitaria de 15 mm en el ventrículo izquierdo. **B** y **C.** Corte axial en el que se aprecia la extensión de la masa descrita y otra adyacente con características de lesión tumoral en la llegada de la vena pulmonar inferior izquierda. La masa era móvil, obliteraba el tracto de salida del ventrículo izquierdo, protruía a través de la válvula aórtica y no tenía plano de separación con el músculo de la pared del tabique. Tenía escaso derrame pericárdico. **D.** Angiorresonancia tridimensional en la que se observa masa intracavitaria del ventrículo izquierdo con infiltración de la pared del tabique y el ápice, la cual atraviesa hacia la aorta a través de la válvula aórtica, generando obstrucción del tracto de salida.

Se diagnosticó angiosarcoma cardiovascular metastásico, y en la junta de especialistas se decidió administrar quimioterapia con ifosfamida, doxorrubicina, mesna y pegfilgrastim. 

## Discusión

Las lesiones quísticas cerebrales son frecuentes y se convierten en un reto diagnóstico, especialmente en los países en desarrollo, como Colombia, donde las enfermedades parasitarias son una de las primeras opciones dado su estatus epidemiológico.

Los angiosarcomas son sarcomas malignos que se originan en las células endoteliales vasculares. Pueden generarse en cualquier región del cuerpo, aunque se encuentran más comúnmente en la piel, las mamas, el hígado y en tejidos profundos y en menos del 5 % de los casos se localizan en la aurícula o el ventrículo izquierdo; en Colombia, no se han informado en edad pediátrica.

La paciente de este caso procedía de una zona con condiciones precarias de salubridad, donde es probable la presencia del complejo teniasis-cisticercosis, que en el humano afecta principalmente el sistema nervioso central. 

La neurocisticercosis es una cestodiasis causada por la larva de *Taenia solium*, la cual se adquiere por el consumo de huevos con embriones del parásito*,* aguas impotables, frutas y vegetales crudos, o directamente por la vía ano-mano-boca de personas con teniasis ^1^. Una vez ingeridos los huevos, en el intestino humano se libera el embrión hexacanto, el cual penetra la pared intestinal y alcanza la circulación sanguínea para luego alojarse en diferentes órganos y tejidos donde se desarrolla el estadio de cisticerco o larva y se forman vesículas únicas o múltiples que pueden permanecen viables durante años ^2^.

En Colombia se han reportado prevalencias de neurocisticercosis de hasta 82,2 % ^3^; sin embargo, su diagnóstico no es sencillo y requiere la combinación de pruebas clínicas, de laboratorio e imagenología, como la tomografía computarizada craneal, la resonancia magnética cerebral, el análisis directo del líquido cefalorraquídeo, la prueba ELISA y el inmunoensayo ^4^^).^

La neurocisticercosis es la enfermedad parasitaria más común del sistema nervioso central y debería considerarse en el diagnóstico diferencial de las metástasis cerebrales, especialmente en pacientes de países endémicos, ya que puede mimetizarse como glioma, abscesos cerebrales o quistes aracnoideos, o parecer metástasis. En la resonancia magnética cerebral, la neurocisticercosis aparece en forma de quistes de pared delgada con edema que, a diferencia de las metástasis, no se realzan con el contraste. El diámetro de las lesiones es menor de 20 mm y a veces se observa un nódulo mural que corresponde al escólex invaginado y constituye un hallazgo patognomónico. El 15 % de los pacientes presenta un único quiste en el sistema nervioso central ^5^.

Pese a la alta sensibilidad de las pruebas serológicas para la detección de antígenos o anticuerpos, una de las grandes desventajas es el elevado porcentaje de reacciones cruzadas, especialmente en la cisticercosis y otras infecciones helmínticas, entre ellas la hidatidosis ^6^. El inmunoensayo tiene un valor predictivo negativo importante y arroja hasta un 40 % de falsos positivos por reacción cruzada con otras teniasis, cestodiasis y tricuriasis. Las pruebas serológicas de la paciente de este caso fueron negativas.

La hidatidosis cerebral es una parasitosis causada por la larva de varias especies del cestodo *Echinococcus,* que se adquiere al consumir huevos con embriones provenientes de alimentos contaminados con heces de animales carnívoros que fungen como huéspedes definitivos ^7^. Una vez ingeridos los huevos de *Echinococcus*, el embrión hexacanto se libera en el intestino delgado humano, atraviesa la pared intestinal y la circulación lo lleva a los órganos, principalmente hígado o pulmón, donde se establece y crece hasta desarrollar quistes de 1 a 15 cm de tamaño.

El diagnóstico de la hidatidosis cerebral se basa en la presentación clínica, los factores de riesgo para el contagio y, primordialmente, el análisis radiológico. En Colombia, las pruebas serológicas para la hidatidosis no están disponibles debido a que esta cestodiasis es relativamente infrecuente, a diferencia de su mayor incidencia en países del cono sur como Argentina, Chile y Uruguay ^8^, países con condiciones medioambientales y ganaderas favorables para la propagación larvaria del *Echinococcus*.

Los quistes hidatídicos intracraneales aislados son poco comunes, y producen síntomas que se manifiestan entre 2 semanas y 6 años, especialmente cefalea y vómito, en el 87 % de los pacientes. En la tomografía computarizada craneal los quistes se ven bien delimitados, con una pared fina, lisa y sin realce con el contraste ^9^^,^^10^.

Una vez se efectúa el retiro completo del quiste por medio de técnicas directas y con base en el estudio de histopatología, es posible llegar a observar las características morfológicas propias del quiste hidatídico: las tres membranas y los protoescólices internos ^11^.

La lesión cerebral detectada en la paciente mediante resonancia magnética simple y contrastada es muy similar a la causada por la infección con *Echinococcus granulosus*, sin por ello descartar la neurocisticercosis; sin embargo, en este caso los resultados negativos del inmunoensayo para cisticercosis descartaron esta parasitosis. La paciente recibió manejo con albendazol a la espera del reporte de patología de las lesiones cerebrales y los nódulos del miembro inferior izquierdo, en el cual se describió una lesión tumoral de alto grado compatible con angiosarcoma.

Con base en este hallazgo se hicieron otros estudios para buscar el sitio primario de la neoplasia y mediante la ecocardiografía y la angiorresonancia cardiaca se halló una masa intracardiaca dependiente del ventrículo izquierdo. El presente caso clínico resalta la importancia de no conformarse con la impresión diagnóstica inicial y hacer uso de todos los métodos diagnósticos al alcance del médico.

El angiosarcoma es una neoplasia muy agresiva, con un alto índice mitótico y capacidad de hacer metástasis por vía hematógena y linfática ^12^. Los angiosarcomas primarios o metastásicos del sistema nervioso central son raros, con una incidencia en adultos de menos del 1 % ^13^. El compromiso del sistema nervioso central se manifiesta con un déficit neurológico múltiple, de rápido inicio y acompañado de hemorragia intracraneana. Cuando el angiosarcoma es cardiovascular, el sitio primario suele ser la aurícula derecha (35 % de los casos) y aparece con metástasis en el sistema nervioso central ^14^. En el caso descrito la paciente tenía el tumor primario en el ventrículo izquierdo, lo que constituye el primer reporte de esta localización según lo encontrado en la búsqueda de publicaciones médicas indexadas.

Según la *American Medical Association*, la incidencia general de los sarcomas cardiacos primarios es de 0,07 % ^15^. En general, el 25 % de los tumores cardiacos son malignos, el 95 % de los malignos son sarcomas, y el 30 % de los sarcomas corresponde a una angiosarcoma primario ^16^. El angiosarcoma es un tumor muy agresivo, el 80 % de los pacientes presenta metástasis en el momento del diagnóstico y el 90 % sobrevive menos de nueve meses después del diagnóstico ^17^. Las lesiones metastásicas generalmente se diagnostican antes del angiosarcoma cardiaco ^16^.

El astrocitoma pilocítico, los ependimomas, los hemangioblastomas y los xantoastrocitomas pleomórficos son algunos de los tumores cerebrales primarios que pueden ser quísticos y remedar una parasitosis intestinal. En una revisión de casos de quistes encontrados mediante neuroimágenes se encontró que las metástasis quísticas cerebrales son relativamente raras, aunque la degeneración quística de las metástasis cerebrales es común en los cánceres de pulmón, seno, páncreas, riñón y en el melanoma ^11^.

El tratamiento convencional del angiosarcoma consiste en la resección quirúrgica, la quimioterapia y la radioterapia ^18^. Con frecuencia, el angiosarcoma está ubicado en órganos vitales y su resección no es factible, como en el caso de nuestra paciente ^19^, por lo que el tratamiento definitivo es solo paliativo ^20^.

El presente reporte de caso llama la atención sobre la necesidad de considerar el angiosarcoma con metástasis cerebral como diagnóstico diferencial de las lesiones quísticas en el sistema nervioso central y de incluirlo en los algoritmos de diagnóstico.
